# Blended-red lighting partially mitigates the cost of light pollution for arthropods

**DOI:** 10.1007/s00442-025-05665-9

**Published:** 2025-01-29

**Authors:** Michela Corsini, Hunter J. Cole, Dylan G. E. Gomes, Kurt M. Fristrup, Jesse R. Barber

**Affiliations:** 1 Department of Biological Sciences, Boise State University, Boise, ID 83725 USA; 2 Institute for Wildlife Studies, Arcata, CA 95518 USA; 3 Current address: Forest and Rangeland Ecosystem Science Center, United States Geological Survey, Seattle, WA 98195 USA; 4 Natural Sounds and Night Skies Division, National Park Service, Fort Collins, CO 80525 USA; 5 Center for Biodiversity and Conservation, American Museum of Natural History, New York, NY 10024 USA

**Keywords:** Light pollution, Arthropods, Invertebrates, Mitigation, Artificial light at night, LED, ALAN

## Abstract

Light pollution disrupts the natural dark–light rhythmicity of the world and alters the spectral composition of the nocturnal sky, with far-reaching impacts on natural systems. While the costs of light pollution are now documented across scales and taxa, community-level mitigations for arthropods remain unclear. To test two light pollution mitigation strategies, we replaced all 32 streetlights in the largest visitor center in Grand Teton National Park (Wyoming, USA) to allow wireless control over each luminaries’ color and brightness. We captured fewer arthropods, across most Orders, in the blended-red light compared to white (3000 K). Interestingly, we found an effect of light brightness and color, suggesting that, overall, more arthropods were attracted by brighter, and white color hues compared to blended-red. Our findings provide valuable insights into the mitigation of artificial light at night, likely one of the primary drivers of global arthropod declines.

**Supplementary Information:**

The online version contains supplementary material available at 10.1007/s00442-025-05665-9.

## Introduction

Anthropogenic light impacts almost one quarter of the planet’s night skies (Falchi et al. 2016) and is burgeoning in brightness by 9.6% every year (Kyba et al. 2023). In addition, the recent development of new lighting technologies has altered the spectral composition of night skies, and older, warmer narrow-spectrum lights (e.g., sodium-vapor lamps emitting yellow light) are giving way to light-emitting diodes (LED) that often produce broader spectrum, bluer light. Most LED lamps are formed by a blue LED that is coated with a phosphor to produce a full-spectrum white light with blue spectral peak. Color temperature is determined by the fraction of the blue light that is converted by the phosphors, high color temperatures being considered ‘cold’, and low color temperatures being considered ‘warm’. This ongoing transition in lighting technologies is being driven by policies aimed at creating energy-efficient lighting systems in the face of climate change (Hölker et al. 2010a).

With over 50% of global residential lighting currently consisting of LEDs (Zissis and Bertoldi 2018), these novel anthropogenic nightscapes disrupt the natural cycles of light within and beyond human settlements, with cascading impacts on biodiversity, from organisms to ecosystems (Hölker et al. 2021; Jägerbrand and Spoelstra 2023). Light pollution disrupts both vertebrate and invertebrate communities by altering their habitat use (McLaren et al. 2018; Spoelstra et al. 2023), migration and orientation (Burt et al. 2023; Lorne and Salmon 2007; Mrosovsky and Carr 1967), distributions (Ciach and Fröhlich 2017; Jägerbrand and Spoelstra 2023), physiology (Boyes et al. 2021; Dominoni et al. 2013), and reproduction (Firebaugh and Haynes 2016; Kriska et al. 1998; Robert et al. 2015). As human development advances, it is imperative that we fully understand the effects of light pollution and find ways to reduce its impact on vulnerable taxa.

Known as the largest and most diverse group within the animal kingdom, arthropods provide key-ecological services (e.g., pollination, pest control, nutrient recycling) and support terrestrial and aquatic trophic webs (Baxter et al. 2005; Noriega et al. 2018; Rader et al. 2016). Their current decline is caused by multiple factors linked to global change (Hallmann et al. 2017), including light pollution (Owens et al. 2020). With more than 60% of arthropods being nocturnal (Hölker et al. 2010b), these animals are clearly likely to be impacted by artificial light at night. Nocturnal moths, for example, are commonly attracted to artificial light (Yamaguchi and Heisenberg 2011) and are declining much faster than sympatric diurnal moths and butterflies (van Grunsven et al. 2020). Researchers reported that the accumulation of moths in lit areas – known as the “vacuum cleaner effect” (Eisenbeis et al. 2006) – creates an ecological sink that removes insects from the immediate, and darker areas located next to the light source. The impacts of light pollution on insects can be observed both instantaneously (Firebaugh and Haynes 2016), and cumulatively after years of light exposure (van Grunsven et al. 2020). These effects extend across life stages [e.g., Lepidoptera larvae and adults (Boyes et al. 2021; van Grunsven et al. 2020)].

Considering the detrimental effects of light pollution on insects, it is crucial that we develop new technologies that minimize the ecological impacts of outdoor lighting (Cole et al. 2021; Longcore et al. 2015). In addition to limiting artificial light use to essential areas and timeframes, mitigation strategies involve adjustments to light spectra (color), brightness (number of photons per unit area), directivity [light orientation, (Dietenberger et al. 2024)], and duration (Antignus 2000). Of particular importance is the growing use of LED luminaires, which allows for all these technological modifications. However, concerns have been raised regarding the increased blue-light emissions of most LED lights, and its negative effects on insects (Pawson and Bader 2014). Blue, short-wavelength light attracts more insects than lights with longer wavelengths [yellow or red (Jägerbrand and Spoelstra 2023)]. Although some studies have not consistently reported these trends (see van Grunsven et al. 2020), the majority indicate that red and yellow attract relatively fewer insects compared to white in most groups (Donners et al. 2018; Longcore et al. 2015; Owens et al. 2020; Seymoure et al. 2024, 2019; Spoelstra et al. 2017). This is primarily because many insects have limited sensitivity to the long wavelength end of the spectrum (Van Der Kooi et al. 2021). Thus, by altering the spectral characteristics of outdoor lighting, we hypothesize that we can reduce the magnitude of insects’ attraction, and efficiently mitigate the ecological costs of light pollution. This is particularly relevant across protected areas, where artificial lighting management can yield superior nocturnal experience for visitors (Fristrup et al. 2024), but its ecological consequences can easily extend beyond the lit areas (Giavi et al. 2020).

Here, we experimentally examined in a unique setting two light pollution mitigation strategies – altered light hue (blended-red vs. 3000K white), and reduced brightness (from 100 to 60%) – to see if these approaches reduce arthropod attraction to light. Previous studies focused on how different light types shaped arthropod activity in urban, peri-urban, and residential areas with pervasive, chronic lighting (Eisenbeis et al. 2006; Longcore et al. 2015; Spoelstra et al. 2023). Our research aimed to evaluate mitigation strategies against artificial light pollution in a natural, protected area setting. The Grand Teton National Park, located in Wyoming, USA, is among the darkest areas on the planet. Consequently, we anticipate a more pronounced response in arthropods to artificial light pollution shifts in this environment, compared to urban areas where chronic exposure to artificial light has led to a reduction in moths’ flight-to-light behavior (Altermatt and Ebert 2016). To examine arthropods’ attraction to light pollution, we converted all 32 streetlights situated in the largest visitor facility in the park to experimental luminaires that let us change light color and brightness wirelessly over two years. Furthermore, to enhance the diversity of arthropod Orders captured, we deployed both passive (flight-intercept traps) and active (UV-bucket trap) sampling methods. Our findings pave the way for a more comprehensive understanding of effective strategies to mitigate the impact of artificial light at night. Long-term studies across diverse environments and encompassing a broader spectrum of arthropods are essential to provide urban planners, landscape managers and, local stakeholders with reliable solutions before implementing costly and potentially inefficient changes to any outdoor illumination system, especially in protected areas.

## Materials and methods

### Study area

**Fig. 1:**
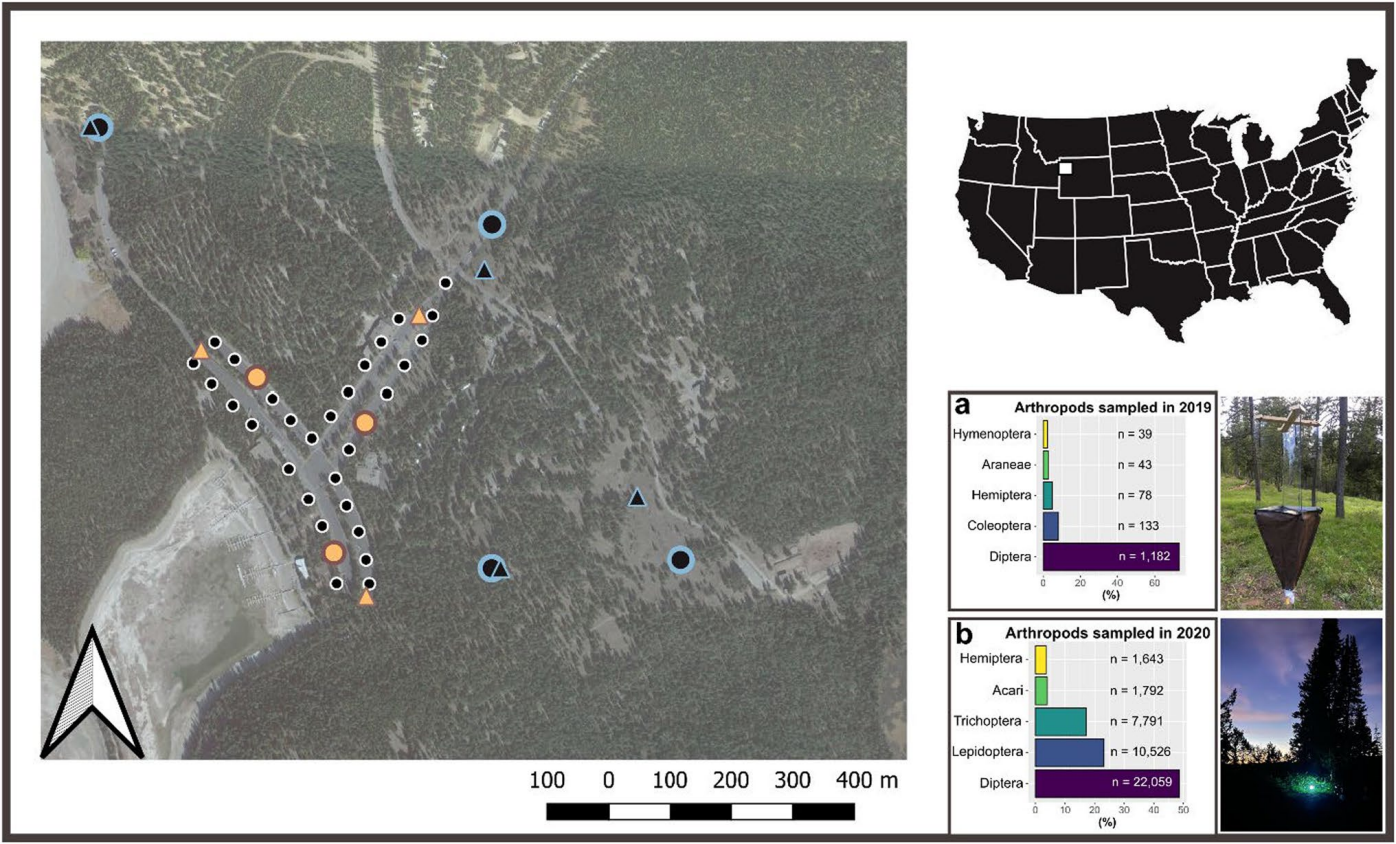
Study area and percentages of the most common arthropods’ Orders collected in 2019 and 2020. Circles and triangles distinguish flight-intercept traps (**a**) from UV-bucket traps’ (**b**) locations. Yellow and blue colors indicate the experimental light presence as lit (yellow) or unlit (blue). The black dots show the locations of the 32 pole-mounted streetlights. The map was generated in QGIS v. 3.34.2. We reprojected the map elements and layers in the local Coordinate Reference System (CRS: NAD83/Wyoming West (ftUS), EPSG:3739) Basic map: ©Google2023. We extrapolated percentages by Order for arthropods sampled using flight-intercept traps in 2019 (**a**), and using UV-bucket traps in 2020 (**b**). Numbers reported on each bar indicate the five prevalent Orders captured each year. We built the barplots based on all the arthropods Orders, including unidentified specimens (see Table S2). Photo credits; a: a flight-intercept trap by Hunter J. Cole, and b: an UV- bucket trap by Clare Throm

We conducted our experiment in Colter Bay Village, Grand Teton National Park (Wyoming, USA; 43°44′28″ N, 110°48′09″ W; Fig. 1). Colter Bay houses the largest visitor center in the park and is defined by a T-shaped parking lot that covers an area of c. 4.3 hectares and includes a grocery store, laundromat, and a marina office, each equipped with different light sources, including small high-pressure sodium, incandescent, and LED bulbs. These localized sources were constant throughout our experiments. Yet, the primary source of artificial light in the parking lot is 32 pole-mounted streetlights (approximately 6 meters high) (Fig. 1). We monitored arthropods at three lit sites within the parking lot and at four adjacent unlit (dark) sites (Fig. 1) in 2019 and in 2020. We selected sampling locations with similar habitat characteristics: that is, dense conifer-forest surrounding clearings of both paved and natural substrates.

### Lighting experiment and arthropod sampling

In June 2019, we replaced the existing 32 streetlights in Colter Bay parking lot (initially consisting of high-pressure sodium vapor lamps, and c. 4000 K white LEDs) with Signify Road Focus Medium (RFM) cobra head luminaires with customized LED-modules and wireless controls. Each RFM luminaire featured three LED modules each, with one module emitting 3400K white light using 16 LEDs with a corresponding temperature of 3400 K. The other two modules emitted blended red-white light as they include an extra full spectrum input for color rendering (e.g., here referred to as “blended-red”) generated using 30 LEDs with a 623 nm peak wavelength (half-power at 614 nm and 629 nm), and two 3000 K white LED. The incorporation of one white LED in each of the red modules provided a small quantity of full-spectrum light that improves human color rendering. Each LED was individually shielded to improve directivity compared to earlier lighting technology. We switched the light colors between white and blended-red and dimmed the light output using Build on Activator ^TM^ (Nedap N. V.), and an outdoor wireless controller (Nedap N.V.). Wireless controls allowed us to switch the Colter Bay nightscape from blended-red to white in either 3-night blocks (2019) or 7-night blocks (2020). In addition, we randomly altered light brightness from 100% to 60% every six nights in 2019. In 2020, we kept light brightness constant at 95%.

**Fig. 2:**
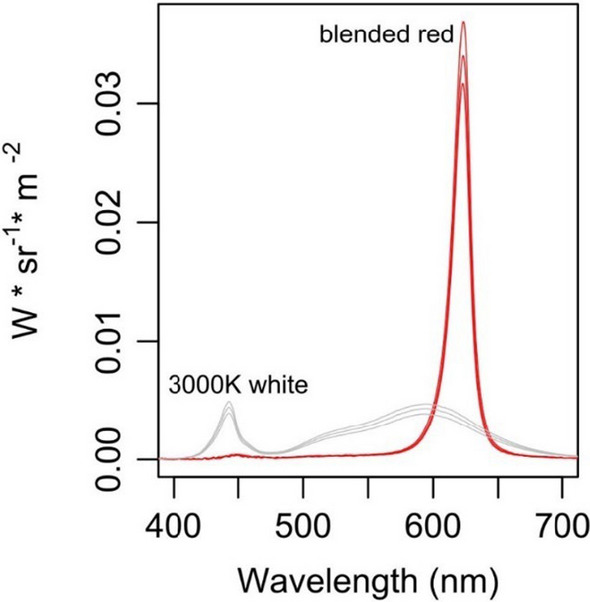
Spectral characteristics of the two light treatments (blended-red and white) we examined in the lighting-experiment set up. Brightness levels are visualized for 60, 80 and 100% in each color option. We obtained the irradiance measurements in a dark room using a spectroradiometer (Stellar Rad blue Wave, Stellar net) placed under the light source at c. 2 m

Light (in terms of spectrum and brightness levels, see Fig. 2), and arthropod trapping systems changed between years: in 2019, we trapped arthropods using flight-intercept traps from June 24^th^ until August 21^st^, barring equipment malfunctions, for a total of 52 nights. We deployed flight-intercept traps before dark and collected arthropods the following day. Due to the low number of arthropods captured with flight-intercept traps in 2019 [especially Lepidoptera—(Fig. 1a)] in 2020 we used UV-bucket traps for a total of 16 experimental nights spanning from July 14^th^ to August 14^th^. Specifically, we deployed UV-bucket traps on the ground (Fig. 1b), and trapped arthropods for two hours after sunset (see Table S1 for specific light color and brightness treatments by experimental night, each year).

### Environmental variables

Considering the influence of moonlight intensity on arthropod activity (Gao et al. 2024; Williams and Singh 1951), we incorporated in the analyses average moonlight intensity for each night of sampling, corresponding to the period of arthropod collection (depending on trap type, thus spanning the whole night for arthropods collected in 2019, and 3 h – from 9.00 pm to 12.00 am – in 2020). We used the R-package *moonlit* v.0.1.0, which accounts for disc brightness, distance to the moon, position of the moon, angle of incidence, and cloud cover. By specifying time and location, moonlit predicts lunar illumination by explaining up to 92% of the variation in illumination levels with a residual standard error of 1.4% compared to 60% explained by moon phase with a residual standard error of 22.6% (Śmielak 2023). We have also included *Ordinal date* (1^st^ of January, recorded as 1, each year) to account for the seasonal shifts in arthropod populations. Moreover, given the influence of temperature on arthropod abundance (Lessard et al. 2011), and flight activity (Chen and Seybold 2014; Taylor 1963), we calculated average temperature (in Celsius) for each night of sampling, corresponding to the time period of arthropod collection (trap type). This spanned from 9.00 pm to 5.00 am of the following day for arthropods collected in 2019, and from 9.00 pm to 12.00 am in 2020. We derived hourly temperature data from USCRN (Diamond et al. 2013). In our study system, wind speed posed a limited concern for arthropod collection during both the sampling periods, as it consistently remained below 6 on the Beaufort scale on our sampling days (Pollard and Yates 1993).

### Statistical analyses

We analyzed the data using R version 4.1.2. (R Development Core Team 2011). Due to the deployment of two distinct arthropod-trapping systems (flight-intercept traps in 2019, and UV-bucket traps in 2020) and different sampling schemes and experimental designs (see Table S1), we conducted separate analyses for each year. Considering the substantial variations in arthropod Orders’ vision (Longcore 2023; Van Der Kooi et al. 2021), we built six distinct models for each of the five most-common arthropod Orders captured by year as well as the combined total number of arthropods sampled to assess their responses to light colors (2019 and 2020) and brightness (2019). Namely, we built 12 distinct Generalized Mixed Effect Models using the *glmmTMB* R-package (Magnusson et al. 2017).

In 2019, we used the total number of arthropods captured in the flight-intercept traps, and the 5 prevalent arthropod Orders as the response variable for each model. In all models, categorical variables included Light color (as red or white), and Experimental light presence (as lit or dark), and the continuous variables included Light brightness (as 60, 65, 70, 75, 80, 85, 90, 95 and 100%), and Averaged moonlight intensity derived from the moonlit package (Śmielak 2023).

In 2020, we fitted the overall number of arthropods and the total number of specimens captured within each Order trapped in UV-bucket traps as response variables. In each of the 6 models, the categorical variables included light color, and experimental light presence, and the continuous variables averaged moonlight intensity, ordinal day, and temperature.

We tested for potential interaction effects between light color and light brightness (in 2019), and between light color and experimental light presence (in both years). We removed interactions if not significant. To account for non-independence of locations sampled multiple times throughout each field season, we included the *sampling location* (*n* = 7) as a random effect (intercept). We applied Z-scored scaling (base R function scale, as (χ_i_ – χ_mean_) / sd) to each numeric predictor for clarity of parameters’ estimates. We finally verified and confirmed the absence of multicollinearity issues using the performance package (Lüdecke et al. 2021): namely, we calculated the variance inflation factors (VIF) in all models, which never exceeded 5. Because the parameters averaged moonlight intensity and ordinal date were strongly correlated (VIF >10), we removed the former from all the models ran in 2020 to avoid potential multicollinearity issues. We visually verified models fit and residuals using the *DHARMa*-package (Hartig 2019). We extracted the predicted model estimates and their respective upper and lower 95% confidence intervals in their original scale by using the *ggeffect* package (Lüdecke 2018), and plotted the outputs with *ggplot2* (Wickham 2011). We built the final figures with Inkscape (Hiitola 2012).

## Results

The UV-bucket traps captured a higher number of arthropods compared to the flight-intercept traps, with a total of 45,412 *versus* 1618 arthropods sampled, respectively (Fig. 1, Table S2). Due to a low number of specimens captured in certain groups, we focused on the five most common Orders collected with each trapping system. Diptera was the most captured Order in both trap types, followed by Coleoptera, Hemiptera, Araneae, and Hymenoptera in flight-intercept traps (2019), and by Lepidoptera, Trichoptera, Acari, and Hemiptera in UV-bucket traps (2020; Fig. 1, Table S2). Data on captures of other Orders is reported in the supplement (Table S2).

**Fig. 3:**
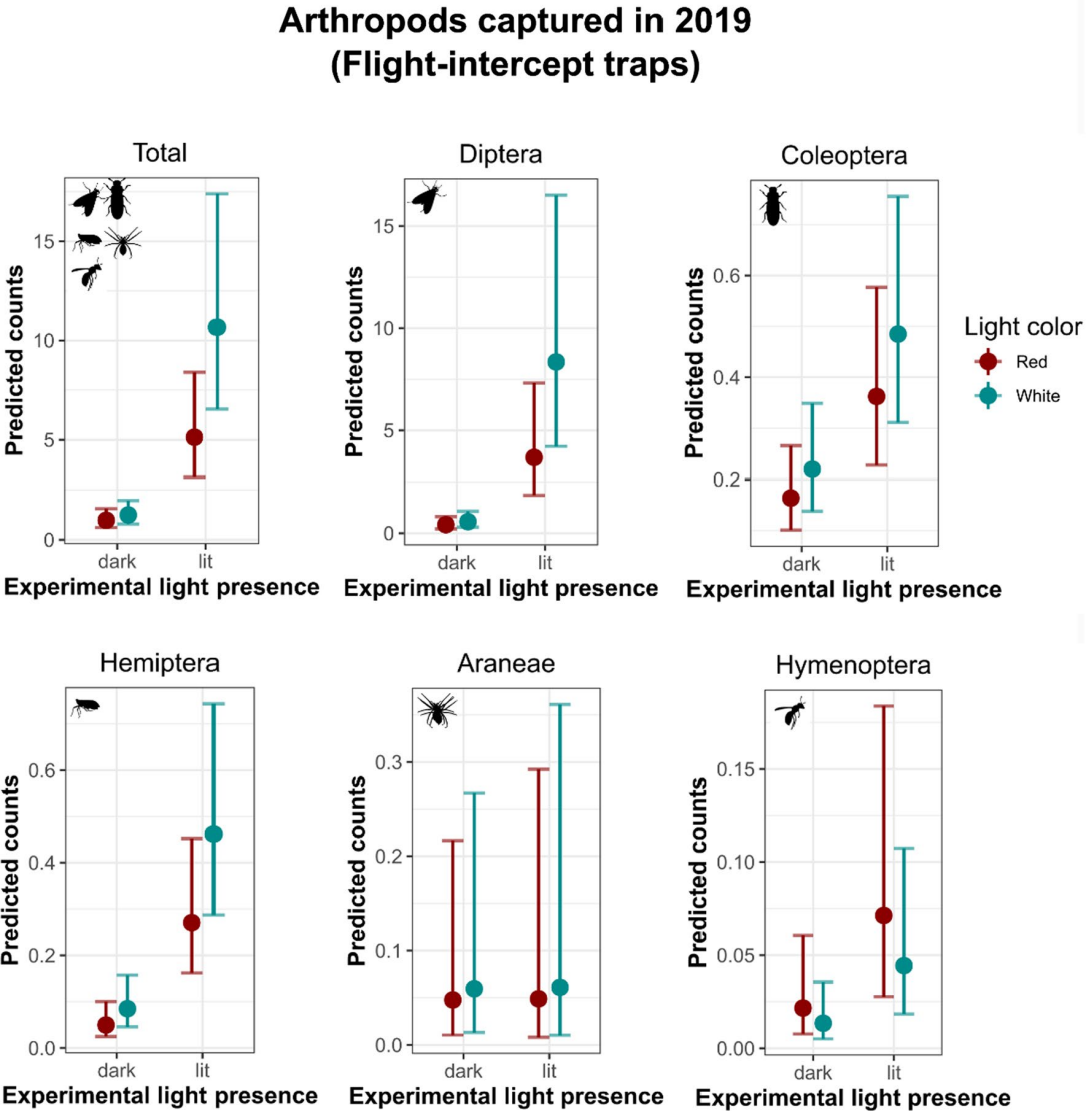
Predicted 2019 arthropod counts and 95% Confidence Intervals derived from negative-binomial, and Poisson distributed generalized linear mixed models by the effect of light color and experimental light presence. We modelled the arthropods sampled in 2019 as a cumulative count (Total) and by Order. Categorical variables included *Light color* (as blended-red or 3000K white), and *Experimental light presence* (as lit or dark). Continuous predictors included *Light brightness* (as %), *Ordinal date*, *Averaged moonlight intensity* (derived from *moonlit*). We fitted *Study site* (*n* = 7) as a random effect in each model to control for non-independence of locations sampled multiple times across the season

In 2019, we found a strong interaction effect between light color and experimental light presence, indicating that, overall, more arthropods were trapped when using brighter light hues, and especially in lit areas. Among all the Orders investigated, we observed a higher number of Diptera being trapped under white light compared to red. Conversely, in dark (unlit) sites, we trapped a lower number of Diptera, Coleoptera, Hemiptera, and Hymenoptera, compared to the lit sites. We did not observe any difference in the abundance of Araneae between different light colors or sites (as lit or dark) (Figs. 2 and 3). Brightness seldom was positively associated with number of Diptera and Araneae captured with flight-intercept traps (Tables S3).

**Fig. 4:**
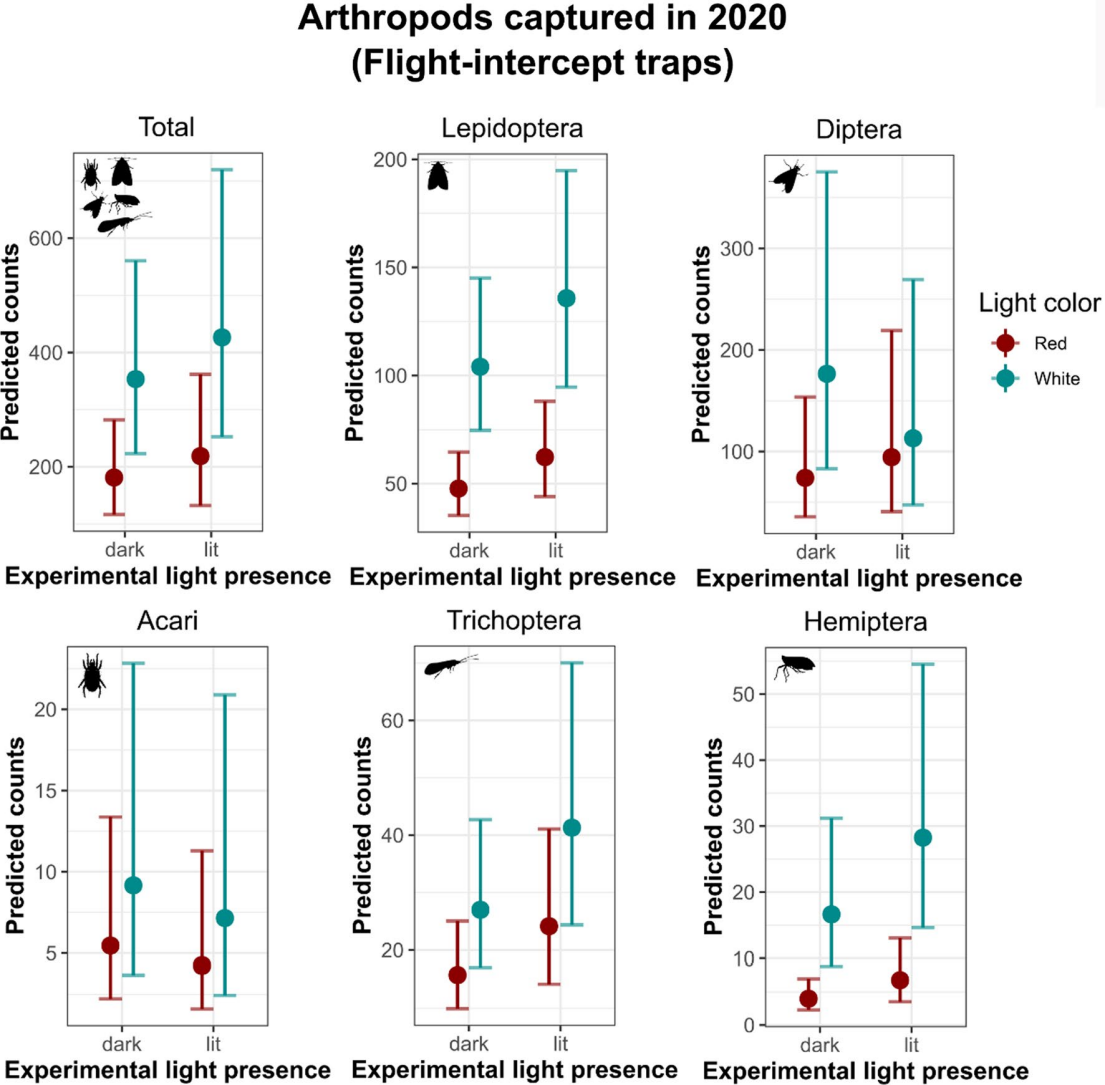
Predicted 2020 arthropod counts and 95% Confidence Intervals derived from negative-binomial or zero-inflated generalized linear mixed models by the effect of light color and experimental light presence. We tested the total number of Arthropods as a cumulative value of all the specimens collected (Total) and individually for the 5 prevalent Orders captured in 2020. Categorical variables included *Light color* (as blended-red or 3000K white), and *Experimental light presence* (as lit or dark). Continuous predictors included *Ordinal date (1= 1*^*st*^* of January)*, and *Averaged moonlight intensity* (derived from *moonlit*). We fitted *Study site* (n = 7) as a random effect in each model to control for non-independence of locations sampled multiple times across the season. Model estimates are reported in supplementary information (Table S4)

In 2020, we also collected a lower total number of arthropods in UV-bucket traps with blended-red light compared to white (Fig. 4, Table S4). When testing arthropod Orders separately, we captured more specimens under 3000 K white light for most of the arthropod Orders investigated, except for Acari (which was likely bycatch, Table S4). With the only exception of Trichoptera – whose number of captured specimens was higher in lit areas – the number of arthropods by Order did not change between illuminated and dark locations when using UV-bucket traps (Fig. 4, Table S4).

In both years and trapping systems there was an important effect of both the seasonal period (ordinal day), and averaged moonlight intensity. Namely, the number of trapped arthropods was generally lower earlier in the season and with increased moonlight intensity levels (Tables S3 and S4).

## Discussion

Our experiment clearly shows that light pollution mitigation strategies, specifically a shift in spectral composition, can protect arthropods. Indeed, by conducting an experiment in the brightest visitor infrastructure of a keystone protected area of the United States, we observed that, overall, blended-red and lower brightness lights attracted fewer arthropods than brighter, 3000 K white light. Besides confirming what previously described in a study conducted on rodents (Longcore et al. 2024), we also provide crucial information to stem the global decline of arthropods in protected areas (Van Klink et al. 2020; Wagner et al. 2021).

Our results align with earlier studies indicating that short wavelength light [but see van Grunsven et al. (2020)], particularly UV, blue, and green lights attract more moths (Brehm et al. 2021; Somers-Yeates et al. 2013), dipterans (Burkett et al. 1998; Deichmann et al. 2021), and hemipterans (Pacheco-Tucuch et al. 2012), than longer wavelength lights (Brehm et al. 2021; van Langevelde et al. 2011; Seymoure et al. 2024). Previous work focusing on white LEDs has shown that Diptera and Trichoptera are strongly attracted compared to dark controls (Carannante et al. 2021). Here, we found more Coleoptera in flight-intercept traps deployed in lit areas compared to the dark ones (Fig. 3, Table S3). This might be explained by either a reduced attraction-to-light behavior which is normally found in insects thriving within light-polluted areas (Altermatt and Ebert 2016; Kaunath and Eccard 2022), or by species-specific responses to light pollution sources, which vary according to the light type and the Coleoptera species. Although further details about coleopteran species diversity in our samples would have greatly helped to explain this trend, the attraction to certain lights is generally attributed to species-specific spectral sensitivity, with red light being generally imperceptible to most nocturnal insects (Longcore 2023; Van Der Kooi et al. 2021). Moreover, many insects use UV fractions of the light spectrum for orientation and navigation, and thus enhance their attraction to lights that emit UV (Froy et al. 2003). Another possible explanation to the reduced number of Coleoptera captured under brighter treatments may also suggest that there is a rapid predation response by bats. Thus, specimens were most likely predated before making it to the trap.

Yet, some arthropods are attracted to warm color temperatures (i.e., yellowish, between 2700 and 3000 K). For instance, vector-dipterans like the phlebotomine sand flies (genus *Sergentomyia*), which can detect light within the yellow, ultraviolet, and blue spectra, can also be lured by red wavelengths (De Felipe et al. 2023; Hoel et al. 2007). Analogously, Bentley et al. (2009) observed a higher abundance of *Anopheles quadrimaculatus* females—a mosquito known as one of the main malaria-vectors—in red (660 nm) light compared to blue (470 nm) light, green (502 nm) light, and the unlit control. Although most LED lights generally attract fewer dipterans than other types of anthropogenic light sources, the data are mixed (Wakefield et al. 2016). There is evidence that the attraction of dipterans to red and orange light may be influenced by background colors. A study on dipterans reported that *Euxesta eluta* was more attracted to green light than yellow and blue when against a white background (Allan 2024). However, when placed against a black background, attraction to lime green was equal to blue and yellow light-traps (Allan 2024). Thus, background colors are a crucial component of visual attraction in insects, and can contribute to a comprehensive understanding of the impact of light pollution (Allan et al. 1987).

Our use of two arthropod trapping systems broadened the range of Orders we examined, enhancing the generalizability of our findings (Fig. 1, Table S2). Between the two trapping systems here used, UV-bucket traps are more appropriate to capture phototactic (i.e., light attracted) night-flying insects which normally belong to Diptera, Hemiptera, Coleoptera, Trichoptera, Hymenoptera, and Lepidoptera (especially moths). Although many factors may influence both abundance and richness of captured specimens (e.g., seasonality, moonlight intensity, habitat type, etc.), the larger number of specimens found in UV-bucket traps compared to flight-intercept traps is almost certainly due to the UV-bulb, which attracts positive-phototactic flying-insects in the trap vicinity (Montgomery et al. 2021). Yet, the flight-to-light behavior rarely goes beyond a 30m-radius in moths (e.g., Truxa and Fiedler 2012), and can be reduced in arthropods found in chronically lit areas like cities (Altermatt and Ebert 2016). Here, the five prevalent arthropod Orders we found in UV-bucket traps were Diptera, Lepidoptera, Trichoptera, and Hemiptera, with Acari (especially mites) captured accidentally, perhaps because of their parasitic-relationship with moths (Treat 1975). The lower number of arthropods captured with flight-intercept traps compared to UV-bucket traps might be also caused by the wind-induced rotation of the traps, which can reduce their efficiency (Montgomery et al. 2021).

Importantly, we found that the flight-intercept traps located in the experimentally lit areas captured more arthropods compared to those in dark locations. This result highlights the effectiveness of passive monitoring over active monitoring when investigating the effects of artificial light pollution on arthropods. In UV-bucket traps, we captured more Diptera, Lepidoptera, Trichoptera, and Hemiptera, under the 3000 K white light. With the sole exception of Trichoptera (see Fig. 4 and Table S4), which showed a higher capture rate in lit locations compared to the dark ones, the lack of important differences in capture rates among the experimental light plots (as lit or dark) for the other Orders may be due to the attraction of additional phototactic flying insects to the UV-bucket traps. Furthermore, when using black lights in brightly lit areas, the black-light competes with the other outdoor lighting at the site (such as the parking lot lights), resulting in either smaller catches or similar catches compared to the dark sites (Eisenbeis et al. 2006). The short distance between lit and unlit sampling locations may also have contributed to similar results in captures while using UV-bucket traps (Table S5). Flight-intercept traps are, instead, a passive form of arthropod-collection (Fig. 1). Moreover, besides the interaction effect between brightness and experimental light presence (as lit or dark), indicating that overall, more arthropods were attracted by brighter light colors in lit areas when using flight-intercept traps, we did not detect any strong effect of light brightness on certain arthropod Orders (including Coleoptera, Hemiptera, and Hymenoptera), and this may be due to either our small sample size or the limited range of brightness levels we tested. The lack of an impact of brightness on certain Orders investigated—here, Coleoptera, Hemiptera, and Hymenoptera—also supports the Steven’s power law’s application to sensory phenomena in insects (Ruchty et al. 2010), where light color becomes more important when the variation in light brightness is less than an order of magnitude (Longcore et al. 2015). Among all Orders, and accordingly with other studies, we captured more spiders when using brighter light hues. Spiders in earlier studies have also been found to be attracted to lights (Heiling 1999; Willmott et al. 2019) possibly benefitting from the higher local abundance of prey especially across urbanized landscapes (Gomes 2020). In contrast, Acari (e.g., mites), did not vary in response to light hue, brightness, or experimental light presence (as lit or dark), further suggesting their presence in the traps as bycatch.

Our experiment showed that lower brightness, and red LEDs are an efficient strategy for mitigating the ecological costs of light pollution on arthropods in Grand Teton National Park, a protected, and generally dark area. Moreover, another study conducted simultaneously with this one revealed that the same red lighting also enhances the overall nocturnal experience of visitors, as it improves visibility of the night sky, increases perception of safety, and aids navigation (Fristrup et al. 2024). The blended-red lighting we used, thus, provides both social and ecological benefits in Grand Teton National Park: one of the darkest areas of the globe. Although certain arthropods—like dipterans—may still be attracted to blended-red LEDs (Bentley et al. 2009; De Felipe et al. 2023), and thus further research is needed to identify potential drivers of this attraction, our research confirmed that changing spectra composition is an efficient strategy in mitigating the ecological costs of light pollution, likely one of the primary drivers of global arthropod decline (Davies and Smyth 2018).

## Supplementary Information

Below is the link to the electronic supplementary material.

Supplementary file1 (DOCX 47 KB)

## Data Availability

Data and codes are available on Dryad Digital Repository,- Corsini (2025)10.5061/dryad.t1g1jwtcv
