# Supplementary material for: Blended-red lighting partially mitigates the cost of light pollution for arthropods
Source: Oecologia. 2025 Jan 29;207(1):26. doi: 10.1007/s00442-025-05665-9 (PMC11779773; doi:10.1007/s00442-025-05665-9)
Supplement: Supplementary file 1 — Supplementary file1 (DOCX 47 KB) [file 442_2025_5665_MOESM1_ESM.docx]

**Supplementary Information**

**Blended-red lighting partially mitigates the cost of light pollution for arthropods**

Michela Corsini*^1^, Hunter J. Cole^1,2^, Dylan G.E. Gomes^1,3^, Kurt M. Fristrup^4^, and Jesse R. Barber^1,5^

^1^ Department of Biological Sciences, Boise State University, Boise, ID, 83725, USA.

^2^ Institute for Wildlife Studies, Arcata, CA, 95518, USA.

^3^ Current address: Forest and Rangeland Ecosystem Science Center, United States Geological Survey, Seattle, WA, 98195, USA.

^4^ Natural Sounds and Night Skies Division, National Park, Service, Fort Collins, CO, 80525, USA.

**^5^** American Museum of Natural History, Center for Biodiversity and Conservation, New York, NY, 10024, USA.

**Abstract**

Light pollution disrupts the natural dark-light rhythmicity of the world and alters the spectral composition of the nocturnal sky, with far-reaching impacts on natural systems. While the costs of light pollution are now documented across scales and taxa, community-level mitigations for arthropods remain unclear. To test two light pollution mitigation strategies, we replaced all 32 streetlights in the largest visitor center in Grand Teton National Park (Wyoming, USA) to allow wireless control over each luminaries’ color and brightness. We captured fewer arthropods, across most Orders, in the blended-red light compared to white (3000 K). Interestingly, we found an effect of light brightness and color, suggesting that, overall, more arthropods were attracted by brighter, and white color hues compared to blended-red. Our findings provide valuable insights into the mitigation of artificial light at night, likely one of the primary drivers of global arthropod declines.

**Keywords**: Light pollution, arthropods, Invertebrates, mitigation, artificial light at night, LED, ALAN

**Table of contents:**

- **Table S1.** Detailed schedules by experimental night illustrating light color (2019 and 2020) and brightness (2019) treatments applied in Colter Bay parking lot. (p.3).
- **Table S2**. Arthropods specimens trapped by Order, year, and trap type. (p.4).
- **Table S3.** Generalized linear mixed models testing the effect of light color (as white or blended-red) and brightness (% wattage) on total number of arthropods and on each of the most common five Orders captured in 2019. (p.5)
- **Table S4.** Generalized linear mixed models testing the effect of light color (as white or blended-red) on the total number of arthropods and on each of the most common five Orders captured in 2020. (p.6)
- **Table S5.** Distances (in m) between the arthropod trap (as flight-intercept trap or UV-bucket trap) and the closest light-pole located in Colter Bay across the seven sampling locations. (p.7)

**Table S1. Detailed schedules by experimental night illustrating light color (2019 and 2020) and brightness (2019) treatments applied in Colter Bay parking lot.**

| **Year** | **2019** | | | | | | | | | | | | | | | | | |
| --- | --- | --- | --- | --- | --- | --- | --- | --- | --- | --- | --- | --- | --- | --- | --- | --- | --- | --- |
| **Month** | **June** | | **July** | | | | | | | | | | **August** | | | | | |
| Treatment interval | 24-26 | 28-30 | 1-3 | 4-6 | 7-9 | 10-11 | 12-14 | 15-18 | 19-21 | 22-24 | 25-26 | 28-30 | 5-6 | 7-9 | 10-12 | 13-15 | 16-18 | 19-21 |
| Light color | Red | White | Red | White | Red | White | Red | White | Red | White | Red | White | White | Red | White | Red | White | Red |
| Light brightness (%) | 100 | 100 | 80 | 80 | 85 | 85 | 65 | 65 | 70 | 70 | 90 | 90 | 80 | 95 | 95 | 60 | 60 | 75 |
| Trapping system | Flight-intercept traps | | | | | | | | | | | | | | | | | |

| **Year** | **2020** | | | | |
| --- | --- | --- | --- | --- | --- |
| **Month** | **July** | | | **August** | |
| Treatment interval | 14-16 | 21-23 | 28-30 | 4-6 | 11-14 |
| Light color | Red | White | Red | White | Red |
| Light brightness (%) | 95 | | | | |
| Trapping system | UV-bucket traps | | | | |

**Table S2.** **Arthropods specimens trapped by Order, year, and trap type.** Orders’ percentages were calculated on all arthropods collected, including the unknown specimens.

| **2019 (Flight-intercept traps)** | | |  | **2020 (UV- bucket traps)** | | |
| --- | --- | --- | --- | --- | --- | --- |
| **Order** | **n** | **%** |  | **Order** | **n** | **%** |
| **Diptera** | 1,182 | 73.05 |  | **Diptera** | 22,059 | 48.58 |
| **Coleoptera** | 133 | 8.22 |  | **Lepidoptera** | 10,526 | 23.18 |
| **Hemiptera** | 78 | 4.82 |  | **Trichoptera** | 7,791 | 17.16 |
| **Araneae** | 43 | 2.66 |  | **Acari** | 1,792 | 3.95 |
| **Hymenoptera** | 39 | 2.41 |  | **Hemiptera** | 1,643 | 3.62 |
| Lepidoptera | 33 | 2.04 |  | Coleoptera | 657 | 1.45 |
| Ephemeroptera | 25 | 1.55 |  | Hymenoptera | 498 | 1.10 |
| Acari | 16 | 0.99 |  | Ephemeroptera | 332 | 0.73 |
| Trichoptera | 5 | 0.31 |  | Thysanoptera | 25 | 0.06 |
| Protura | 4 | 0.25 |  | Neuroptera | 19 | 0.04 |
| Thysanoptera | 3 | 0.19 |  | Araneae | 17 | 0.04 |
| Collembola | 1 | 0.06 |  | Pseudoscorpion | 4 | 0.01 |
| Unknown | 56 | 3.46 |  | Psocoptera | 3 | 0.01 |
| **Total** | **1,618** | **100** |  | Collembola | 2 | 0 |
|  |  |  |  | Orthoptera | 2 | 0 |
|  | | |  | Dermaptera | 1 | 0 |
|  |  |  |  | Unknown | 36 | 0.08 |
|  |  |  |  | **Total** | **45,412** | **100** |

**Table S3.** **Generalized linear mixed models testing the effect of light color (as white or blended-red), brightness (%), and experimental light presence (as lit or dark) on arthropod communities (in total and by Order) trapped with flight-intercept traps in 2019.** We used a negative binomial error distribution in all models except for Araneae, and Hymenoptera, where we used a Poisson-error distribution. Significance levels are reported **in bold:** ***p<0.05, **p<0.01, ***p<0.001**.

| **Total arthropods** | | | | |
| --- | --- | --- | --- | --- |
| **Parameters** | **Estimate** | **se** | **z-value** | **p-value** |
| Intercept | -0.016 | 0.236 | -0.070 | 0.944 |
| **Light brightness _sc_ * Experimental light presence (lit)** | **0.492** | **0.196** | **2.520** | **0.012*** |
| Light color (white) | 0.240 | 0.157 | 1.530 | 0.126 |
| Light brightness (%) _sc_ | 0.007 | 0.006 | 1.150 | 0.250 |
| **Experimental light presence (lit)** | **1.650** | **0.344** | **4.790** | **<0.001***** |
| Averaged moonlight intensity _sc_ | 1.680 | 1.746 | 0.960 | 0.336 |
| Ordinal date _sc_ | -0.016 | 0.085 | -0.180 | 0.855 |
| **Temperature _sc_** | **0.068** | **0.019** | **3.540** | **<0.001***** |
| *Error distribution: negative binomial* | | | | |
| **Diptera** | | | | |
| **Parameters** | **Estimate** | **se** | **z-value** | **p-value** |
| Intercept | -0.871 | 0.336 | -2.590 | 0.009** |
| **Light color (white) * Experimental light presence (lit)** | **0.523** | **0.252** | **2.070** | **0.038*** |
| Light color (white) | 0.297 | 0.214 | 1.390 | 0.164 |
| **Light brightness (%) _sc_** | **0.014** | **0.007** | **2.020** | **0.043*** |
| **Experimental light presence (lit)** | **2.172** | **0.485** | **4.480** | **<0.001***** |
| Averaged moonlight intensity _sc_ | 3.782 | 2.133 | 1.770 | 0.076 |
| Ordinal date _sc_ | -0.040 | 0.102 | -0.390 | 0.696 |
| Temperature _sc_ | 0.033 | 0.023 | 1.440 | 0.151 |
| *Error distribution: negative binomial* | | | | |
| **Coleoptera** | | | | |
| **Parameters** | **Estimate** | **se** | **z-value** | **p-value** |
| Intercept | -1.808 | 0.245 | -7.370 | <0.001*** |
| Light color (white) | 0.292 | 0.214 | 1.360 | 0.173 |
| Light brightness (%) _sc_ | -0.009 | 0.013 | -0.760 | 0.447 |
| **Experimental light presence (lit)** | **0.792** | **0.283** | **2.800** | **0.005**** |
| Averaged moonlight intensity _sc_ | -3.148 | 3.809 | -0.830 | 0.408 |
| **Ordinal date _sc_** | **-0.849** | **0.221** | **-3.830** | **<0.001***** |
| **Temperature _sc_** | **0.152** | **0.045** | **3.360** | **<0.001***** |
| *Error distribution: negative binomial* | | | | |
| **Hemiptera** | | | | |
| **Parameters** | **Estimate** | **se** | **z-value** | **p-value** |
| Intercept | -3.000 | 0.356 | -8.430 | <0.001*** |
| Light color (white) | 0.534 | 0.322 | 1.660 | 0.097 |
| Light brightness (%) _sc_ | -0.007 | 0.021 | -0.340 | 0.735 |
| **Experimental light presence (lit)** | **1.694** | **0.345** | **4.910** | **<0.001***** |
| Averaged moonlight intensity _sc_ | -2.990 | 5.973 | -0.500 | 0.617 |
| Ordinal date _sc_ | 0.476 | 0.303 | 1.570 | 0.117 |
| Temperature _sc_ | 0.107 | 0.068 | 1.590 | 0.112 |
| *Error distribution: negative binomial* | | | | |
| **Araneae** | | | | |
| **Parameters** | **Estimate** | **se** | **z-value** | **p-value** |
| Intercept | -3.043 | 0.772 | -3.940 | <0.001*** |
| Light color (white) | 0.222 | 0.310 | 0.710 | 0.475 |
| **Light brightness (%) _sc_** | **0.039** | **0.019** | **2.030** | **0.043*** |
| Experimental light presence (lit) | 0.025 | 1.122 | 0.020 | 0.982 |
| Averaged moonlight intensity _sc_ | 10.011 | 5.811 | 1.720 | 0.085 |
| **Ordinal date _sc_** | **-0.607** | **0.292** | **-2.080** | **0.038*** |
| Temperature _sc_ | 0.085 | 0.065 | 1.300 | 0.192 |
| *Error distribution: Poisson* |  |  |  |  |
| **Hymenoptera** | | | | |
| **Parameters** | **Estimate** | **se** | **z-value** | **p-value** |
| Intercept | -3.835 | 0.526 | -7.290 | <0.001*** |
| Light color (white) | -0.474 | 0.374 | -1.270 | 0.206 |
| Light brightness (%) _sc_ | -0.036 | 0.023 | -1.570 | 0.117 |
| **Experimental light presence (lit)** | **1.194** | **0.436** | **2.740** | **0.006**** |
| Averaged moonlight intensity _sc_ | -11.453 | 7.004 | -1.640 | 0.102 |
| **Ordinal date _sc_** | **2.669** | **0.493** | **5.420** | **<0.001***** |
| Temperature _sc_ | 0.178 | 0.095 | 1.870 | 0.061 |
| *Error distribution: Poisson* | | | | |

**Table S4.** **Generalized Linear Mixed Models testing the effect of Light color (as white or blended-red) and experimental light presence (as lit or dark)** **on arthropod communities (in total and by Order) trapped with UV-bucket traps in 2020.** Experimental light presence (as lit or dark) and Light color (as blended-red or white) were fitted as categorical predictors, Ordinal date (1^st^ of January = 1) and averaged temperature were scaled (sc) and fitted as continuous predictors. Significance p-values are reported **in bold:** ***p<0.05, **p<0.01, ***p<0.001**.

| **Total arthropods (n = 106)** | | | | |
| --- | --- | --- | --- | --- |
| **Parameters** | **Estimate** | **se** | **z-value** | **p-value** |
| Intercept | 5.200 | 0.223 | 23.320 | <0.001*** |
| **Light color (white)** | **0.668** | **0.151** | **4.420** | **<0.001***** |
| Experimental light presence (lit) | 0.188 | 0.327 | 0.570 | 0.570 |
| **Ordinal date _sc_** | **1.710** | **0.238** | **7.190** | **<0.001***** |
| **Temperature _sc_** | **0.143** | **0.035** | **4.050** | **<0.001***** |
| *Error distribution: negative binomial* | | | | |
| **Lepidoptera (n = 106)** | | | | |
| **Parameters** | **Estimate** | **se** | **z-value** | **p-value** |
| Intercept | 3.866 | 0.152 | 25.360 | <0.001*** |
| **Light color (white)** | **0.780** | **0.141** | **5.550** | **<0.001***** |
| Experimental light presence (lit) | 0.266 | 0.218 | 1.220 | 0.220 |
| **Ordinal date _sc_** | **1.028** | **0.206** | **4.990** | **<0.001***** |
| **Temperature _sc_** | **0.134** | **0.032** | **4.260** | **<0.001***** |
| *Error distribution: negative binomial* |  |  |  |  |
| **Diptera (n = 106)** | | | | |
| **Parameters** | **Estimate** | **se** | **z-value** | **p-value** |
| Intercept | 4.304 | 0.368 | 11.700 | <0.001*** |
| **Light color (white**)* **Experimental light presence (lit)** | **-0.689** | **0.325** | **-2.120** | **0.034*** |
| **Light color (white)** | **0.869** | **0.219** | **3.980** | **<0.001***** |
| Experimental light presence (lit) | 0.243 | 0.562 | 0.430 | 0.665 |
| **Ordinal date _sc_** | **1.805** | **0.288** | **6.270** | **<0.001***** |
| **Temperature _sc_** | **0.133** | **0.042** | **3.190** | **0.001**** |
| *Error distribution: negative binomial* | | | | |
| **Acari (n = 106)** | | | | |
| **Parameters** | **Estimate** | **se** | **z-value** | **p-value** |
| Intercept | 1.698 | 0.452 | 3.760 | <0.001*** |
| Light color (white) | 0.518 | 0.364 | 1.420 | 0.154 |
| Experimental light presence (lit) | -0.248 | 0.637 | -0.390 | 0.697 |
| **Ordinal date _sc_** | **3.519** | **0.681** | **5.160** | **<0.001***** |
| Temperature _sc_ | 0.065 | 0.096 | 0.670 | 0.501 |
| *Error distribution: negative binomial* | | | | |
| **Trichoptera (n = 106)** | | | | |
| **Parameters** | **Estimate** | **se** | **z-value** | **p-value** |
| Intercept | 1.975 | 0.303 | 6.520 | <0.001*** |
| **Light color (white)** | **0.960** | **0.239** | **4.020** | **<0.001***** |
| **Experimental light presence (lit)** | **1.186** | **0.422** | **2.810** | **0.004**** |
| **Ordinal date _sc_** | **4.487** | **0.377** | **11.880** | **<0.001***** |
| **Temperature _sc_** | **0.232** | **0.051** | **4.560** | **<0.001***** |
| *Error distribution: zero- inflated* | | | | |
| **Hemiptera (n = 106)** | | | | |
| **Parameters** | **Estimate** | **se** | **z-value** | **p-value** |
| Intercept | 1.384 | 0.282 | 4.910 | <0.001*** |
| **Light color (white)** | **1.427** | **0.262** | **5.440** | **<0.001***** |
| Experimental light presence (lit) | 0.530 | 0.406 | 1.310 | 0.190 |
| **Ordinal date _sc_** | **2.352** | **0.528** | **4.460** | **<0.001***** |
| Temperature _sc_ | -0.087 | 0.078 | -1.120 | 0.260 |
| *Error distribution: negative binomial* | | | | |

**Table S5**. Distances (in m) between the arthropod trap (as flight-intercept trap or UV-bucket trap) and the closest light-pole located in Colter Bay across the seven sampling locations. Distances were measured in qGIS using the line-measure tool.

| **Location ID** | **Experimental light presence** | **Distance (m) _intercept traps_** | **Distance (m) _UV-bucket traps_** |
| --- | --- | --- | --- |
| CORA1 | Dark | 508.41 | 450.91 |
| CRNR1 | Dark | 199.54 | 214.13 |
| DAL01 | Dark | 394.74 | 402.66 |
| NOR01 | Dark | 120.75 | 65.84 |
| SHLA1 | Lit | 0 | 21.47 |
| AMLA1 | Lit | 0 | 24.86 |
| GSLA1 | Lit | 0 | 21.10 |
